# Road Traffic Injury Is an Escalating Burden in Africa and Deserves Proportionate Research Efforts

**DOI:** 10.1371/journal.pmed.0040170

**Published:** 2007-06-26

**Authors:** Emmanuel Lagarde

## Abstract

Changing the mindset of road users in Africa will be a challenge, says the author, but many lives are at stake.

Research into road safety in developing countries is scarce, especially in Africa. This is inconsistent with the size of the problem: it has been predicted that by 2020, road traffic injuries will rank as high as third among causes of disability-adjusted life years (DALYs) lost [1]. While South-East Asia has the highest proportion of global road fatalities (one-third of the 1.4 million occurring each year in the world), the road traffic injury mortality rate is highest in Africa (28.3 per 100,000 population when corrected for under-reporting, compared with 11.0 in Europe [2]). I selected African countries with recent available data on road mortality and number of vehicles in use (Table 1). When comparing death per 10,000 vehicles, the contrast appears even more stark, with 1.7 deaths per 10,000 vehicles in high-income countries across the world [3] and more than 50 in low-income African countries.

**Table 1:**
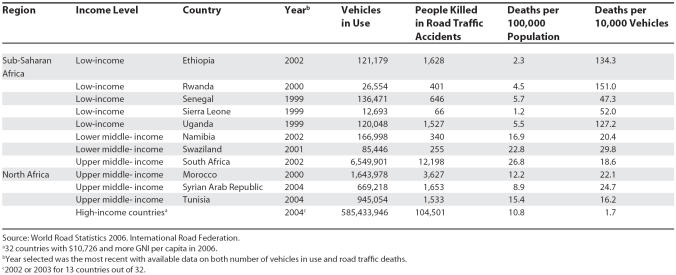
Traffic Fatality Indicators in Africa

In addition, while developing countries already account for more than 85% of all road traffic deaths in the world [4–6], the upsurge in the number of vehicles per inhabitant will result in an anticipated 80% increase in injury mortality rates between 2000 and 2020 [2]. In Africa, it has been estimated that 59,000 people lost their lives in road traffic crashes in 1990 and that this figure will be 144,000 people by 2020, a 144% increase [7]. By contrast, developed countries have experienced a decreasing trend since the 1960s.

Because road traffic injuries have long been considered to be inevitable and caused by random, unpredictable events, the international community's response to this worldwide public health crisis came relatively late. The World Health Organization (WHO) arranged a consultation meeting in April 2001, which led to a report entitled “A 5-year WHO strategy for road traffic injury prevention” that summarises the main recommendations from the working group [8]. In 2003, the United Nations Secretary-General sounded the alarm with an official statement [9] describing the global public health challenge posed by road traffic injuries and encouraging Member States to address the problem. One of the recommendations is to promote and facilitate research on this subject, especially in low-income countries where knowledge gaps often jeopardise appropriate resource allocation. Much needs to be done. Global research and development funding for road traffic injuries were estimated in 1996 to range from US$24 to US$33 million, compared with more than US$900 million for HIV/AIDS [1]. Moreover, the overwhelming majority of this money is spent in developed countries.

I reviewed published studies from Africa in the field of road traffic injuries to identify pending priorities for future research programmes that would enable the promotion of effective public health policies in road safety.

## Trends in Road Traffic Injuries in Africa

The number of vehicles per inhabitant is still low in Africa: less than one licensed vehicle per 100 inhabitants in low-income Africa versus 60 in high-income countries. Fleet growth leads to increased road insecurity in developing countries [10–12]. This explains, for example, the reported 400% increase in road deaths in Nigeria between the 1960s and the 1980s [13]. Available historical data from developed countries show that it is only when a development threshold is achieved that the road mortality starts to decrease [7,14,15]. Such a threshold is far from being reached in sub-Saharan Africa. Indeed, in South Africa, the most developed African country, there were already 17 licensed vehicles per 100 inhabitants in 2005, and no decline in road traffic deaths has been observed so far [16].

## Crash and Casualty Characteristics

A comprehensive literature review published in 1997 showed that pedestrians accounted for between 41% and 75% of all road traffic deaths in developing countries [17]. In Africa, pedestrians and passengers of public transportation are the most affected [17]. They represented 80% of all road traffic deaths in Kenya in 1990 [18] and 67% of all road traffic injuries as recorded in Ghana in the 1989–1991 period [19]. Pedestrians alone accounted for 55% of road traffic deaths in Mozambique in the 1993–2000 period [20], and 46% of road traffic deaths in Ghana between 1994 and 1998 [21]. This large proportion of vulnerable road users is explained by a traffic mix of incompatible users (pedestrians, cyclists, motorbikes, cars, and trucks) with, for example, communities living within the vicinity of roads or the lack of pavement along large urban streets.

The severity of road traffic crashes is also likely to be much greater in Africa than anywhere else, because many vulnerable road users are involved, but also because of the poor transport conditions such as lack of seat belts, overcrowding, and hazardous vehicle environments. Death/injury ratios are, however, not easy to compare because of the differential reporting bias for fatal and non-fatal injuries.

## Surveillance

The paucity of surveillance data from African countries leads to uncertainties, and probably to major under-estimates of the size of the problem [22]. Implementing an effective and sustainable information system related to road traffic injuries should be among the first priorities, as this enables us to address three decisive objectives. First, the monitoring of trends in road traffic injuries is a unique tool to assess the effectiveness of new prevention policies. Second, such a tool also allows for a useful account of the characteristics of traffic insecurity, helping the prioritisation of effective interventions (identification of hot spots, vulnerable road users, regional variations, and so on). Third, issuing persuasive figures through regular national or regional reports will help advocate for the allocation of appropriate resources. Current efforts from many African countries need to be encouraged and evaluated, and success stories should be replicated. Data can come from death statistics [23,24], hospital registries [18,20,25,26], and even from population surveys [27–30]. Collection of police reports is, however, the most frequent system implemented in several African countries [20,21,31–35]. These initiatives need to be generalised, standardised, and, most importantly, evaluated and optimised accordingly.

## Pre-Hospital and Hospital Emergency Care

In Ghana, a study conducted in a rural area showed that most injured people are transported to hospitals staffed by general practitioners with no training in trauma care [36]. Even in urban centres, mortality is high because of disorganisation and lack of adequate equipment or trained personnel [37–39]. Limitations that explain the poor outcome for people involved in road traffic crashes in Africa have been identified, and include lack of trained surgeons, intensive care staff, and field paramedics; underserved medical facilities; inappropriate dedicated transportation; and disorganised or non-existent emergency and trauma services.

Improvement in this area is not an easy task. Even a developed country like the United States needed several years to achieve effectiveness of its trauma care system [40]. There are examples of successful cost-effective interventions implemented in low-income countries, both for pre-hospital care [41,42] and hospital care [43], and a consensus is now emerging about the need for a training package for trauma teams designed for use in Africa. Evaluation of such a program in Uganda has been reported recently [44]. It is therefore necessary to carefully assess which low-cost interventions that can be implemented in African countries are most likely to be effective in improving recovery or survival among persons with life-threatening injuries.

## Prevention

Research efforts enabling progress in primary and secondary prevention involve several fields: the human factor, including behaviours and driving capacities, vehicle conditions, and infrastructure. Although those topics are relevant to road safety all over the world, there are specificities in Africa that need to be taken into account.

**Figure:**
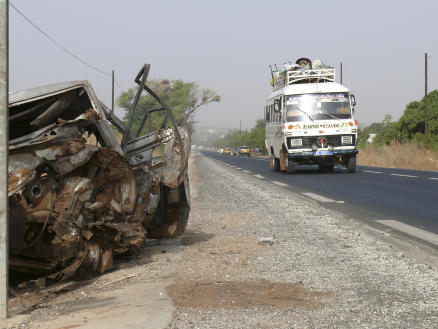


African public perception of the risks of road traffic injury must be understood in order to be able to adapt and apply prevention campaigns that have proved successful elsewhere. Because research in this field has never been a priority, almost nothing is known. Most available results are from South Africa and are concerned with superstition of cab drivers [45], perception of accident causes [46], youth risk behaviours [47], and psychosocial impact of road traffic injuries on drivers and relatives [48]. A study from Tanzania [49] has compared perceptions in rural and urban areas, and points out the key role of the media. In regards to actual prevention campaigns, I found one study reporting the perceptions of 50 professional drivers in Ghana of a campaign using TV spots [50].

The risk associated with drinking and driving has been thoroughly described in developed countries, but is certainly also a key determinant in developing countries, as shown in Kenya [51], Nigeria [52,53], Tanzania [54], and South Africa [55]. The Kenyan study is particularly interesting as it shows that alcohol not only plays a major role in road crashes involving 4-wheeled vehicles, but also plays a significant role for 2-wheeled vehicles and pedestrians [51].

Speed control probably carries the greatest potential to save lives. The key factor in the effectiveness of traffic regulations is the drivers' perception that they run a high risk of being detected and punished for infractions [56]. Unfortunately, in Africa, the combination of a low enforcement level, frequent corruption of police officers, and low public awareness dooms any traffic regulation, including speed control, to failure. A report from Ghana illustrated how, in recognition of the shortcomings of enforcement, speed bumps and rumble strips were installed and reduced fatalities by 55% [57].

Almost everything remains to be done in the field of medical driving incapacities. I found only two studies on the subject from Africa, related to visual impairment of drivers in Nigeria [58] and South Africa [59]. The potentially high prevalence of drivers' incapacities calls for intense research efforts to identify and prioritise medical conditions responsible for substantial numbers of road traffic injuries.

Even though the impact of vehicle condition has almost never been scientifically assessed [60], the issue was recognised early as a priority for traffic safety. Two problems have been identified in this respect: lack of maintenance and importation of old vehicles. In most African countries, there is no inspection requirement, and whenever legally mandated safety standards are advocated they have been ruled out, as it is considered that vehicle owners cannot afford the corresponding buying and maintenance costs. As far as importations are concerned, several African countries have instituted laws limiting or banning importation of the oldest vehicles, but applying them is often demanding. In Senegal, for example, a law was promulgated in 2001 which banned importation of cars older than five years and trucks older than 10 years, but the law only started to be applied in 2003.

Finally, road infrastructures are also a component of road safety and are often prioritised by governments and funding agencies. However, scientific evaluation studies are missing [61], mainly because of the lack of appropriate surveillance accident data. Current infrastructure interventions are often inappropriate, as it is impossible to localise and prioritise points of intervention [31].

## Road Safety and Development

As recently advocated by Khayesi and Peden [22], road safety in Africa is “part of the broader development process”. The situation is particularly worrying in this continent because of the combination of incompatible road users, poor vehicle condition, under-developed infrastructure, lack of risk awareness, and ineffective enforcement jeopardised by corruption or bribery. The road transport system is the dominant form of inland transportation and carries more than 95% of passenger traffic. This sector is often prioritised in donor development plans in countries such as Cameroon [62], Ghana [63], Gabon [64], and Senegal [65], to cite only a few African countries receiving European Union development aid. Road transportation is essential to access markets and services, and to unlock agricultural potential, which will lead to improved incomes in rural areas. Road safety concerns could also endanger income from tourism. Foreign affairs departments are increasingly cautioning their nationals against road injury risk, just as with infectious disease risk [66]. In a study of 794 German tourists who had travelled to Kenya, Tanzania, Senegal, The Gambia, India, Nepal, Thailand, or Brazil, more than 5% experienced an accident [67].

## Past Research Efforts

**Figure 1:**
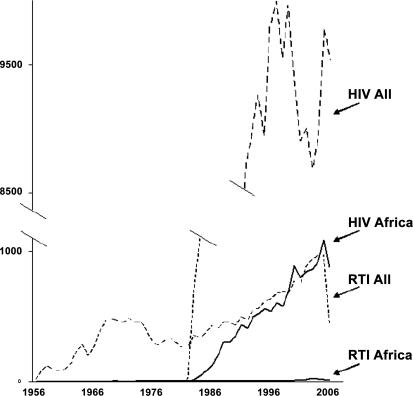
Number of References Related to Road Traffic Injury and HIV/AIDS, from the PubMed Database (Figure: A. Flores) RTIs, road traffic injuries

In order to gauge the magnitude of past research efforts in road traffic injuries from Africa, I performed an automatic search in the PubMed database with the MeSH (a medical indexing system) term “accidents, traffic” and found a total of 25,320 references. Of these, only 290 were selected by adding the MeSH term “Africa”. For the purpose of comparison, I performed the same search with MeSH term “HIV” and found 193,695 references, of which 12,674 related to Africa. Figure 1 shows how these figures evolved between 1956 (the first year for which a related citation was found in PubMed) and 2006. Clearly, the number of publications related to Africa has been increasing only slightly since the late 1980s, while publications from all over the world have escalated exponentially, from about 100 per year in the 1960s to almost 1,000 per year today. Only the comparison of curves is of interest here, as many studies related to road traffic safety (and to HIV/AIDS) have been published in journals not referenced in PubMed.

An Ad Hoc Committee on health research was convened in 1996 by WHO and provided estimates of research and development expenditures for major global health problems using a capture–recapture method [1]. In 1990, expenditure per one DALY lost was US$85 for HIV/AIDS and US$0.83 for road traffic injuries (the ratio is 102).

## Conclusion

Many results on road injury prevention are available from developed countries. We now urgently need to scale up surveillance and research efforts in developing countries in order to determine how to build on these results [68,69], taking regional specificities into account. A proper surveillance system should produce reliable data handled by trained epidemiologists to issue intervention priorities. Indispensable research actions include the assessment of population knowledge, attitudes, and behaviours; identification of regional intervention priorities; effectiveness and cost-effectiveness evaluations of interventions; and evaluation of care management practices and training procedures.

In Africa, driving a car is still considered a privilege, an enviable option, not a risky task with inherent responsibilities. Unfortunately, Africa has other burning public health priorities. Documented success stories in road safety are needed to demonstrate that road traffic accidents need not be inevitable and unpredictable, but are avoidable. Changing the mindset of road users will be a challenge, but many lives are at stake.

## Supporting Information

Alternate Language Article S1 Translation of the manuscript into French by EL (201 KB DOC).

## Abbreviations

DALY: disability-adjusted life year
WHO: World Health Organization
