# Supplementary material for: Road Traffic Injury Is an Escalating Burden in Africa and Deserves Proportionate Research Efforts
Source: PLoS Med. 2007 Jun 26;4(6):170. doi: 10.1371/journal.pmed.0040170 (PMC1896192; doi:10.1371/journal.pmed.0040170)
Supplement: Alternate Language Article S1 — (201 KB DOC). [file pmed.0040170.sd001.doc]

La part grandissante de l’insécurité routière en Afrique doit entraîner des efforts de recherche proportionnés.

**Word count : 2990**

Emmanuel Lagarde

INSERM, U593, Equipe Avenir Santé et Insécurité Routière, Bordeaux, F-33000 France

Université Victor Segalen Bordeaux 2, Case 11

146 rue Léo Saignat

33076 Bordeaux Cedex

France

Tel: +(33) 5 57 57 15 04

Fax: +(33) 5 57 57 45 28

email: emmanuel.lagarde@isped.u-bordeaux2.fr

Malgré son importance en terme de mortalité et de morbidité, l’insécurité routière fait l’objet de peu d’attention dans les pays en développement, en particulier en Afrique. D’ici à 2020, les traumatismes causés par les accidents de la route occuperont la troisième place parmi les causes mondiales d’années de vie perdues ajustées sur l’incapacité (DALYs) [1]. Alors que le sud-est asiatique représente la plus grande part des décès sur la route (un tiers des 1,4 million survenant chaque année dans le monde), les taux de mortalité routière sont les plus élevés en Afrique (28,3 pour 100 000 lorsque la sous-déclaration est prise en compte, à comparer à 11.0 pour 100 000 en Europe [2]). Nous présentons dans le tableau 1 une sélection de pays Africains disposant de données récentes sur la mortalité routière et le nombre de véhicules en service. Lorsque l’on compare les décès pour 10 000 véhicules, les contrastes sont plus importants, avec 1.7 décès pour 10 000 véhicules dans les pays à hauts revenus [3] contre plus de 50 dans les pays d’Afrique à bas revenus.

De plus, alors que les pays en développement représentent déjà plus de 85% des décès sur la route dans le monde [4,5,6], l’accroissement du nombre de véhicules par habitant va entraîner une augmentation de 80% de la mortalité routière entre 2000 et 2020 [2]. En Afrique, on estime qu’environ 59 000 personnes ont perdu la vie dans un accident de la circulation en 1990 et que ce chiffre pourrait être proche de 144 000 en 2020, correspondant à une augmentation de 144% [7]. Par comparaison, les pays développés connaissent une décroissance de cette mortalité depuis les années 60.

**Tableau 1.** Indicateurs de mortalité routière en Afrique.

|  | **Année** | **Véhicules** | **Tués dans un accident de la route** | **Décès pour 100 000** | **Décès pour 100 000 véhicules** |
| --- | --- | --- | --- | --- | --- |
| **Afrique Sub-Saharienne** |  |  |  |  |  |
| **Bas revenus** |  |  |  |  |  |
| Ethiopie | 2002 | 121 179 | 1 628 | 2,3 | 134,3 |
| Rwanda | 2000 | 26 554 | 401 | 4,5 | 151,0 |
| Sénégal | 1999 | 136 471 | 646 | 5,7 | 47,3 |
| Sierra Leone | 1999 | 12 693 | 66 | 1,2 | 52,0 |
| Ouganda | 1999 | 120 048 | 1 527 | 5,5 | 127,2 |
|  |  |  |  |  |  |
| **Revenus intermédiaires-bas** |  |  |  |  |  |
| Namibie | 2002 | 166 998 | 340 | 16,9 | 20,4 |
| Swaziland | 2001 | 85 446 | 255 | 22,8 | 29,8 |
|  |  |  |  |  |  |
| **Revenus intermédiaires-hauts** |  |  |  |  |  |
| Afrique du Sud | 2002 | 6 549 901 | 12 198 | 26,8 | 18,6 |
|  |  |  |  |  |  |
| **Afrique du Nord** |  |  |  |  |  |
| Maroc | 2000 | 1 643 978 | 3 627 | 12,2 | 22,1 |
| Syrie | 2004 | 669 218 | 1 653 | 8,9 | 24,7 |
| Tunisie | 2004 | 945 054 | 1 533 | 15,4 | 16,2 |
|  |  |  |  |  |  |
| **Pays à hauts revenues**^*^ | 2004^**^ | 585 433 946 | 104 501 | 10,8 | 1,7 |

Source : World Road Statistics 2006. International Road Federation.

* 32 pays avec 10726 USD et plus de revenus par habitant en 2006

** 2002 or 2003 pour 13 des 32 pays

$ L’année sélectionnée est celle la plus récente pour laquelle des données sont disponibles à la fois pour le nombre de véhicules et la mortalité.

Parce que la mortalité routière causée par des événements imprévisibles et aléatoires a longtemps été considérée comme inévitable la réponse de la communauté internationale à cette crise sanitaire mondiale est arrivée tardivement. L’Organisation Mondiale de la Santé a organisé en avril 2001 une consultation conduisant à un rapport intitulé “5-year WHO strategy for road traffic injury prevention”, qui résume les recommandations principales du groupe de travail [8]. En 2003, le Secrétaire Général des Nations Unies a tiré la sonnette d’alarme dans une déclaration officielle [9] décrivant l’enjeu de santé publique global que représente les traumatismes consécutifs aux accidents de la circulation et encourageant les pays membres de s’impliquer dans la résolution de ce problème. L’une de ces recommandations était de promouvoir et faciliter la recherche, en particulier dans les pays pauvres dans lesquels le manque de connaissances compromet souvent la bonne répartition des ressources disponibles. Il y a beaucoup à faire : il a été estimé que les financement de recherche et développement dans ce domaine était de 24 à 33 millions de dollars, à comparer avec plus de 900 millions de dollars pour le VIH/SIDA [1]. Bien entendu, la majeure partie de ces fonds est dépensée dans les pays développés

Nous avons effectué une revue de la littérature des études conduites en Afrique dans le domaine des accidents de la circulation afin d’identifier les priorités actuelles susceptibles de guider les futurs programmes de recherche nécessaires à la mise en place de politiques de sécurité routière efficaces.

**Evolution de la morbidité et de la mortalité routière en Afrique.**

Le nombre de véhicules par habitant est encore bas en Afrique : moins d’un véhicule immatriculé pour 100 habitants contre 60 pour 100 habitants dans les pays à hauts revenus. L’accroissement du parc automobile entraîne mécaniquement une augmentation de la mortalité routière [10,11,12]. Ceci explique, par exemple, le saut de 400% enregistré au Nigeria entre les années 60 et les années 80 [13]. Les données historiques disponibles en provenance des pays développés montrent que ce n’est qu’à partir d’un certain seuil de développement que la mortalité routière commence à décliner [7,14,15]. Un tel seuil est loin d’être atteint en Afrique au sud du Sahara. En effet, même en Afrique du Sud, le pays Africain le plus développé, on enregistrait 17 véhicules immatriculés pour 100 habitants en 2005 et aucune baisse de la mortalité n’a été observée jusqu’à maintenant [16]. Kopits et Cropper ont proposé une estimation des évolutions de la mortalité par accidents de la route dans chaque région du monde en utilisant un modèle basé sur le lien entre le revenu par habitant et la mortalité routière : 59 000 personnes ont perdu la vie sur les routes en Afrique au sud du Sahara en 1990 et ce chiffre augmentera vraisemblablement de 144% jusqu’en 2020 pour atteindre 144 000 personnes. A l’inverse, les pays à hauts revenus ont enregistré 123 000 décès sur la route en 1990 et 80 000 sont estimés pour 2020. [7].

**Caractéristiques des victimes et des accidents en Afrique**

Une revue exhaustive de la littérature publiée en 1997 montre que les piétons représentaient de 41% à 75% de l’ensemble des décès dans les pays en développement [17]. En Afrique, piétons et passagers des transports publiques sont les plus touchés [17]. Ils représentaient 80% de l’ensembles des décès par accident de la route au Kenya en 1990 [18], 67% des victimes au Ghana entre 1989 et 1991 [19]. Les piétons représentaient à eux seuls 55% des décès au Mozambique dans la période 1993-2000 [20], et 46% au Ghana entre 1994 et 1998 [21]. Cette proportion importante d’usagers vulnérables s’explique par la cœxistence sur la route d’usagers incompatibles, tels ces villages ou ces quartiers situés le long de voies à grande circulation, où la présence de piétons sur les chaussées urbaines due à l’absence de trottoirs.

La gravité des accidents est également beaucoup plus grande en Afrique que partout ailleurs, de par le nombre d’usagers vulnérables mais aussi des conditions de transports : absence de ceintures, surpopulation, structures des véhicules agressives.

Les rapports du nombre de décès au nombre de victimes ne sont cependant pas faciles à comparer du fait d’un taux de déclaration différent pour les morts et les blessés.

**Surveillance**

La faiblesse des données de surveillance en provenance des pays Africains conduit à des incertitudes, probablement une sous-estimation de l’ampleur du problème [22]. La mise en place d’un système de surveillance efficace et pérenne est une priorité qui doit permettre d’atteindre trois objectifs : d’abord la connaissance des évolutions de l’accidentalité est nécessaire pour évaluer l’efficacité des politiques de prévention ; deuxièmement, un tel outil fourni une description des caractéristiques locales de l’insécurité routière, à même d’identifier les priorités d’intervention (localisation des points noirs, des usagers vulnérables, des variations régionales …) , enfin la publication de chiffres fiables au moyen, par exemple, de rapport réguliers, permettant de mobiliser les ressources appropriées. Les efforts actuels de plusieurs pays Africain doivent être encouragés et évalués. Les expériences réussies doivent être répliquées. Les données recueillies aujourd’hui proviennent soit des statistiques de décès [23,24], des registres hospitaliers [18,20,25,26], ainsi que d’enquêtes menées en population [27,28,29,30]. Le recueil de rapports de police constitue cependant le système de surveillance le plus fréquent, mis en place dans plusieurs pays d’Afrique [20,21,31,32,33,34,35]. Ces initiatives doivent être généralisées, standardisées et optimisées.

**Prise en charge hospitalière et pré-hospitalière**

Au Ghana, une étude conduite dans une zone rurale a montré que la plupart des bléssés de la route sont conduits dans des centres de santé dépourvus de personnels disposant d’une formation spécifique sur la prise en charge des traumatisés [36]. Même dans les centres urbains, la mortalité est élevée à cause de problèmes d’organisation et du manque de professionnels formés et correctement équipés [37,38] [39]. Les contraintes principales qui expliquent la faiblesse des résultats obtenus sont ainsi identifiées : manque de chirurgiens, d’unités de soins intensifs, de personnels de terrain, de moyens de transport adaptés et absence ou faiblesse des services d’urgences et de traumatologie.

Les améliorations dans ce domaine ne sont pas aisées. Même un pays développé comme les Etats-Unis a eu besoin de plusieurs années pour disposer d’un système efficace de prise en charge des traumatisés [40]. Il y a cependant des exemples d’interventions coût-efficaces réussies dans les pays à bas revenus, qu’il s’agisse de prise en charge pré-hospitalière [41,42] ou des soins hospitaliers [43]. Un consensus émerge maintenant sur le besoin d’un cursus de formation à destination des équipes de traumatologie, adapté aux contraintes des pays en développement. Une étude évaluant un tel dispositif en Ouganda a été publiée récemment [44]. Il est ainsi nécessaire d’identifier avec soin quelles sont les interventions peu coûteuses qui peuvent être mises en place dans les pays d’Afrique et qui permettront d’améliorer les chances de rétablissement et de survie des personnes ayant subis des traumatismes.

**Prévention**

Les efforts de recherche permettant des progrès en prévention primaire et secondaire s’inscrivent dans plusieurs champs : le facteur humain, (comportements et capacités de conduite), l’état des véhicules et l’infrastructure. Même si ces thèmes ont un sens pour la sécurité routière dans tous les pays du monde, il existe des spécificités en Afrique qu’il faut identifier, comprendre et prendre en compte.

Il est nécessaire de décrire et comprendre la perception des risques d’accidents de la circulation des divers groupes de population en Afrique de façon à être capable d’adapter et d’appliquer les mesures de prévention qui ont prouvé leur efficacité ailleurs. Parce que la recherche dans ce domaine n’a jamais été une priorité, rien ou presque rien n’est connu. La plupart des résultats disponibles proviennent d’Afrique du Sud, sur les superstitions des chauffeurs de taxis [45], la perception des causes d’accidents [46], les comportements à risque des jeunes [47], l’impact psychologique des traumatismes sur les victimes et leur entourage [48]. Une étude conduite en Tanzanie [49] a comparé les perceptions dans des zones urbaines et rurales et montrent le rôle prépondérant des médias. Quant aux campagnes de prévention elles-mêmes, nous n’avons trouvé qu’une étude relative à la perception de spots télévisés chez 50 conducteurs professionnels au Ghana [50].

Les risques associés à la conduite sous l’emprise de l’alcool ont été bien décrits dans les pays développés mais constituent certainement un déterminant clef dans les pays Africains, comme cela a été montré au Kenya [51], au Nigeria [52,53], en Tanzanie [54] et en Afrique du Sud [55]. L’étude Kenyane est particulièrement intéressante car elle démontre que l’alcool joue un rôle majeur dans les accidents impliquant les véhicules à quatre roues ainsi que dans les accidents impliquant les deux-roues et les piétons [51].

Le contrôle de la vitesse est une mesure qui porte probablement le plus fort potentiel en terme de vies sauvées. Le facteur clef de l’efficacité d’un code de la route est la perception du conducteur d’un fort risque d’être arrêté et puni lorsqu’il le viole [56]. Malheureusement, en Afrique, la combinaison d’une pression de contrôle-sanction faible, la corruption fréquente des forces de police et le faible niveau d’information des conducteurs compromettent les règles qui régissent la conduite routière, et en particulier les limitations de vitesse. Une étude conduite au Ghana illustre comment, prenant acte de l’échec du contrôle-sanction, les autorités ont opté pour la mise en place de dos d’ânes et de bandes rugueuses qui ont permis de réduire de 55% la mortalité sur les axes concernés [57].

Tout reste à faire dans le domaine des incapacités médiales à la conduite en Afrique. Nous n’avons trouvé que deux études sur ce thème, relatives aux problèmes de vision au Nigeria [58] et en Afrique du Sud [59]. La prévalence des incapacités des conducteurs est probablement élevée et des efforts importants de recherche doivent être accomplis pour identifier les pathologies et handicaps responsables du plus grand nombre d’accidents.

Même si l’impact de l’état des véhicules n’a jamais été mesuré de manière scientifique [60], le problème a été identifié très tôt comme une priorité pour la sécurité routière avec deux composantes principales : le manque d’entretien et l’importation de vieux véhicules. Dans la plupart des pays Africains, aucune obligation n’existe en matière d’inspection, et lorsque le sujet des normes de sécurité est abordé, il est souvent mis de coté, considérant que les propriétaires de véhicules ne pourront pas en assumer les coûts. En ce qui concerne les importations, plusieurs pays ont institué des lois limitant ou interdisant l’importation des véhicules les plus vieux mais leur application est souvent difficile. Au Sénégal, par exemple, une loi a été promulguée en ce sens en 2001, interdisant l’importation des automobiles de plus de 5 ans et des poids lourds de plus de 10 ans, mais elle n’a été appliquée qu’en 2003.

Enfin, les infrastructures routières jouent également un rôle dans la sécurité routière, et cela constitue souvent la priorité des gouvernements et des bailleurs de fonds. Les études dans ce domaine sont cependant rares [61], principalement faute de données de surveillance : les infrastructures existantes sont souvent inadaptées et les priorités d’intervention ne sont pas identifiées [31].

**Sécurité routière et développement**

Comme cela a été dit par Khayesi et Peden [22], la sécurité routière en Afrique fait partie d’un processus de développement plus global. La situation est particulièrement préoccupante dans ce continent à cause du partage de la route par des usagers incompatibles, du mauvais état des véhicules, des infrastructures insuffisantes, de la faible perception des risques et de la faible efficacité du contrôle-sanction, compromise par la corruption. La route constitue le système de communication dominant, transportant 95% des voyageurs. Ce secteur est souvent considéré comme une priorité par les programmes de développement comme au Cameroun [62], au Ghana [63], au Gabon [64], et au Sénégal [65], pour ne citer que quelques pays destinataires de fonds en provenance de l’Union Européenne. Le transport routier est incontournable pour l’accès au marché des biens et services, et rendre disponibles les ressources agricoles pour l’exportation afin d’améliorer les revenus des populations rurales. Les problèmes d’insécurité routière peuvent également compromettre les ressources touristiques. Les ministères des affaires étrangères attirent de plus en plus l’attention de leurs ressortissants sur le risque d’accidents de la route dans certains pays, comme ils le font pour les maladies infectieuses [66]. Dans une étude de 794 touristes allemands rentrant d’un voyage au Kenya, en Tanzanie, au Sénégal, en Gambie, en Inde, au Népal, en Thaïlande ou au Brésil, plus de 5% avaient eu un accident de la route [67].

**Les efforts de recherche**

Afin d’estimer l’ampleur des efforts de recherche sur les accidents de la circulation en Afrique, nous avons procédé à une recherche dans la base de données bibliographiques PubMed avec l’expression (MeSH term) “accidents, traffic” et trouvé 25 320 références. Parmi celles-ci, seules 290 ont été sectionnées en ajoutant le mot-clef “Africa”. Par comparaison, nous avons effectué la même recherche avec le mot-clef “HIV” et trouvé 193 695 références, parmi lesquelles 12 674 comportaient le mot « Africa ». La figure 1 montre comment ces chiffres ont évolué entre 1956 (la première année pour laquelle nous avons trouvé une publication) et 2006. On observe que le nombre de publications relatives aux accidents de la circulation en Afrique augmente légèrement à partir de la fin des années 80 alors que l’ensemble des publications sur les accidents a connu une croissance exponentielle de 100 par an dans les années 60 à presque 1000 par an aujourd’hui. Remarquons que seule la comparaison des courbes n’a de sens ici car de nombreuses études sur les accidents de la circulation (et sur le VIH/SIDA) sont publiées dans des revues qui ne sont pas référencées dans PubMed.

Un comité Ad Hoc sur la recherche en santé organisé en 1996 par l’OMS a fourni des estimations des dépenses de recherche et développement pour les problèmes de santé principaux en utilisant la méthode de capture-recapture [68]. Les dépenses pour une année de vie perdue ajustée sur l’incapacité (DALY) étaient, en 1990, de 85 USD pour le VIH/SIDA et de 0,83 USD pour les traumatismes routiers (un rapport de 1 à 102).

**Conclusion**

De nombreux résultats de recherche sont disponibles dans les pays développés. Il faut maintenant porter nos effort sur la surveillance et la recherche dans les pays en développement de façon à déterminer comment tirer parti des connaissances disponibles ailleurs [69] [70] et de manière urgente. Un système de surveillance efficace doit pouvoir produire des données fiables exploitées par des épidémiologistes disposants d’une formation adaptée leur permettant de dégager les priorités en tenant compte des spécificités régionales d’interventions. Parmi les actions de recherche indispensables, citons l’étude des connaissances, attitudes et comportements des populations, l’identification des priorités d’interventions locales, les évaluations économiques des interventions et l’évaluation des prises en charge et des procédures de formations des personnels médicaux.

En Afrique, conduire un véhicule est considéré comme un privilège, un comportement enviable, pas comme un risque comportant des responsabilités. Malheureusement, l’Afrique a d’autres priorités importantes de santé publique. Les témoignages d’actions ayant prouvé leur efficacité sont nécessaires pour montrer qu’il est possible d’éviter les accidents, qu’ils ne constituent pas une fatalité. Changer les mentalités des usagers est une tâche immense devant laquelle il ne faut pas renoncer car de nombreuses vies sont en jeu.

**Figure 1**. Nombre de références relatives aux accidents de la circulation et au VIH/SIDA dans la base de donnée PubMed.
